# Digestible Indispensable Amino Acid Scores (DIAAS) of Six Cooked Chinese Pulses

**DOI:** 10.3390/nu12123831

**Published:** 2020-12-15

**Authors:** Fei Han, Paul J. Moughan, Juntao Li, Shaojie Pang

**Affiliations:** 1 Academy of National Food and Strategic Reserves Administration (Former Name: Academy of State Administration of Grain), Beijing 100037, China; psj@ags.ac.cn; 2 Riddet Institute, Massey University, Palmerston North 4442, New Zealand; p.j.moughan@massey.ac.nz; 3 State Key Laboratory of Animal Nutrition, College of Animal Science and Technology, China Agricultural University, Beijing 100193, China; lijuntao@cau.edu.cn

**Keywords:** Chinese pulses, DIAAS, true ileal protein digestibility, amino acids, protein quality, pig, human

## Abstract

Values for the digestible indispensable amino acid score (DIAAS) of a protein are based on true ileal amino acid (AA) digestibility values obtained in adult humans or in the growing pig as an animal model. An experiment was conducted using growing pigs to determine the true ileal digestibility (TID) values of AA in six cooked Chinese pulses (kidney bean, mung bean, adzuki bean, broad beans, peas and chickpeas). Each pulse was included in a diet as the only source of crude protein (CP). An N-free diet was given to allow determination of gut endogenous AA losses. Seven growing pigs each fitted with a T-cannula at the terminal ileum were allotted to a 7 by 6 incomplete Latin square with seven diets and six 7-d periods. The true digestibility values % for the total indispensable AA were higher (*p* < 0.001) for broad beans (87.3 ± 2.98) and lower (*p* < 0.001) for kidney bean (73.3 ± 4.84) than for the other pulses. For the older child (over 3 years), adolescent and adult, the DIAAS (%) was 88 for kidney bean, 86 for mung bean, 76 for chickpeas, 68 for peas, 64 for adzuki bean and 60 for broad beans.

## 1. Introduction

The common pulse comprises many different varieties and is the largest legume crop with an annual production of over 31 million tons worldwide [1]. Of the pulses, the kidney bean, mung bean, adzuki bean, peas and chickpeas are the most commonly consumed in China [2]. Pulses are generally characterized as rich sources of complex carbohydrates, vitamins and minerals, including folate, potassium and iron, in addition to having low lipid content [3,4]. They also contain significant amounts of protein (22–30% by weight) and are important plant-based sources of protein [5]. Especially in developing regions, cereal grains and pulses are often the main component of the human diet, and provide a large proportion of the dietary protein intake [6], with pulses providing proportionally more protein than the cereals. In comparison with cereal grains, pulses have higher protein contents. Generally, consumers are showing an increased interest in plant-based sources of protein, but accurate data on their digestible amino acid compositions is sparse. 

An accurate assessment of the dietary protein quality and amino acid (AA) digestibility of food products are important [7,8], and the digestible indispensable amino acid score (DIAAS) has been endorsed by the Food and Agriculture Organization of the United Nations as the preferred method for determining protein nutritional quality [9,10]. A current barrier to the widespread use of DIAAS, however, is the limited amount of published data available for the true ileal AA digestibility of human foods [10] and especially for foods in developing regions of the world. Ileal amino acid digestibility refers to measurement of digestibility at the end of the small intestine and replaces the less accurate measure of fecal digestibility [11] while true digestibility denotes that the digestibility measure has been corrected for the potentially confounding endogenous amino acids found in ileal digesta [12]. To calculate DIAAS, it is necessary to determine the true ileal digestibility (TID) of each AA, which is preferably determined in humans, however, if this is not possible, the growing pig is a suitable animal model for protein digestion in the adult human [9,13]. The physiology of protein digestion to the end of the small intestine is similar between the adult human and growing pig [14] and the growing pig model has been shown to give true ileal amino acid digestibility values not statistically significantly different from those determined in the adult human [15]. Therefore, the objective of the present study was to determine the apparent and true ileal AA digestibility values and DIAAS values of six cooked pulses commonly consumed in China using the growing pig as an animal model for protein digestibility in the adult human. The novelty of the present study is that true ileal amino acid digestibility was determined in pulses, prepared as for human consumption.

## 2. Materials and Methods 

The animal use protocols were reviewed and approved by the China Agricultural University Animal Care Committee (AW07040202-1, Beijing, China). All procedures were performed strictly according to the Guide for Experimental Animals of the Ministry of Science and Technology (Beijing, China).

### 2.1. Sample Procurement and Preparation

Kidney bean, mung bean, adzuki bean, broad beans, peas and chickpeas were sourced for the study. Kidney bean (Variety name: *Xiyun No.2*) and mung bean (Variety name: *Yulv No.1*) were purchased from the Shanxi Province. Adzuki bean (Variety name: *Jihong No.16*), broad beans (Variety name: *Qingcan No.14*), peas (Variety name: *Dingwan No.4*) and chickpeas (Variety name: Xinying *No1 Y2-12*) were purchased from Hebei, Qinghai, Gansu and Xingjiang Provinces, respectively. The cooking mimicked the common method of cooking pulses for human consumption, used in China. All of the ingredients were washed and soaked for 12 h with 25 °C deionized water, and then cooked in added water (20% of dry weight of raw pulses) for 70 min using a commercially available cooker (passing 100 °C steam). All the cooked materials were dried (hot air circulation) and ground through a 2 mm mesh prior to use in the experimental diets (Table 1).

### 2.2. Animals Experiment and Diets

Each pulse was included in a diet as the sole source of crude protein (CP) and AA such that the diets were iso-nitrogenous based on tabulated CP contents of the ingredients (Table 2). An N-free diet was used to determine the basal ileal endogenous N losses. Vitamins and minerals were included in all diets to meet or exceed current requirement estimates for growing pigs [16]. Titanium dioxide was included in all diets (0.3%) as an indigestible marker for calculating the ileal digestibility of AA [17].

Seven growing pigs (castrated boars, (Duroc × Landrace) × Yorkshire; initial body weight: 33.7 ± 1.44 kg) each fitted with a T-cannula at the terminal ileum according to the method of Stein et al. [18] were allotted to a 7 by 6 incomplete Latin square with seven diets (6 pulses and protein-free) and six 7-d periods [19]. No pig received the same diet more than once during the study and there were, therefore, six independent replicates per diet. The growing pig was adopted as an animal model for protein digestion in the adult human. All pigs were individually housed in stainless-steel metabolism crates (1.4 m by 0.9 m by 0.7 m) with adjustable sides in an environmentally controlled metabolism room (22 °C ± 2.5 °C and 12 h of light and 12 h of dark). Humidity varied from 55% to 65% during the experiment. A feeder and a nipple drinker were installed in each pen. After 14 d of recovery from surgery for implantation of the T-cannulas, pigs were fed the experimental diets. At the end of the study, pigs had a body weight of 47.5 ± 3.16 kg.

The experimental diets were given to the pigs at a level of 8% of metabolic body weight (BW^0.75^) (kg) in 2 equal meals daily (07:30 h and 16:30 h). The amount of food supplied to each pig was recorded for each period. After an adaptation period of 5 days for each diet, ileal digesta were collected for 9 h daily (from 08:00 to 17:00 h) on days 6 and 7. The digesta collection lasted for 9 h daily beginning at 08:00 h according to the protocol described by Stein et al. [18]. Plastic bags were attached to the barrel of the cannulas and removed whenever they were filled with digesta and immediately stored at −20 °C to prevent bacterial degradation of the AA in the digesta. At the end of the experiment, digesta samples were thawed, mixed by pig and period, sub-sampled and lyophilized in a vacuum-freeze dryer (Tofflon Freezing Drying Systems, Minhang District, Shanghai, China). All pigs had ad libitum access to water via a drinking nipple [17].

### 2.3. Chemical Analysis

Before chemical analysis, ingredient, diet and digesta samples were ground through a 1-mm screen and mixed thoroughly. Ingredient, diet and ileal digesta samples were analyzed for AA (AOAC method 982.30 E [a, b, c]) [20]. For methionine and cysteine the samples were subjected to cold performic acid oxidation overnight and then hydrolyzed with 7.5 N HCl at 110 °C for 24 h before AA determination. Tryptophan was determined after hydrolyzing the sample with LiOH for 22 h at a constant temperature of 110 °C. The concentration of titanium in the diets and ileal digesta samples was determined using the method described by Short et al. [21]. All analyses were conducted in duplicate. Dry matter (DM) and CP were determined according to standard methods GB/T 6435-2014 [22] and GB/T 6432-2018 [23].

### 2.4. Data Analysis

The endogenous ileal AA flows were determined for pigs fed the protein-free diet [24,25,26].

Apparent and true ileal AA digestibility values were calculated using the following equations (units are g/kg DMI) [25,26]:

AIDAA (%) = 100 − ((AAdigesta/AAdiet) × (Tidiet/Tidigesta)) × 100 (1)

where AIDAA is the apparent ileal digestibility of AA, AAdigesta is the concentration of AA in the ileal digesta DM, AAdiet is the concentration of AA in the diet DM, Tidiet is the concentration of Ti in the diet DM and Tidigesta is the concentration of Ti in the ileal digesta DM.

TIDAA = AID + ((IAAend/AAdiet) × 100) (2)

where IAAend is the ileal endogenous AA losses.

DIAA reference ratios were calculated.

DIAA reference ratio

(3) $$=\;\frac{\mathrm{mg}\;\mathrm{of}\;\mathrm{the}\;\mathrm{digestible}\;\mathrm{dietary}\;\mathrm{indispensable}\;\mathrm{AA}\;\mathrm{in}\;1\;g\;\mathrm{of}\;\mathrm{the}\;\mathrm{test}\;\mathrm{protein}}{\mathrm{mg}\;\mathrm{of}\;\mathrm{the}\;\mathrm{dietary}\;\mathrm{indispensable}\;\mathrm{AA}\;\mathrm{in}\;1\;g\;\mathrm{of}\;\mathrm{the}\;\mathrm{reference}\;\mathrm{protein}}$$

Three indispensable amino acid reference requirement profiles were chosen for calculation of DIAAS: Birth to 6 months, 0.5 to 3 year-old-child and the older child, adolescent and adult profile [9].

DIAAS was calculated using the following equation [9,16]:

DIAAS (%) = 100 × lowest value of the DIAA reference ratio (4)

### 2.5. Statistical Analysis

The data were analyzed using the Proc GLM model of SAS 9.4 statistical software. The pig was the experimental unit, and pig and period were random effects and diet was the fixed effect. LSMEANS were calculated. Statistically significant (*p* < 0.05) differences in amino acid digestibility among diets were determined using orthogonal contrasts.

## 3. Results

### 3.1. CP and AA Compositions of the Six Cooked Pulses

All pigs remained healthy throughout the study and readily consumed the diets. The total gross AA concentrations of the six cooked pulses on an as-fed basis ranged from 18.4% (adzuki bean) to 25.4% (broad beans; Table 3). The gross CP contents of the cooked pulses on an as-fed basis ranged from 24.3% (peas) to 29.7% (broad beans). Compared with the other indispensable AA, all cooked pulses contained relatively high amounts of leucine and lysine and low amounts of tryptophan and methionine.

### 3.2. Mean Apparent Ileal Digestibility (AID) of CP and AA in the Six Cooked Pulses

The AID of CP in broad beans and peas were greater (*p* < 0.001) than that in kidney bean, mung bean and adzuki bean (Table 4). The mean AID of the indispensable AA in broad beans, adzuki bean and peas was greater (*p* < 0.001) than that in kidney bean and mung bean. The AID values of most AAs in broad beans were not different (*p* > 0.05) from those in peas, except that the AID of serine in broad beans was greater than that in peas (*p* < 0.001). The AID values of most AAs in broad beans were not different (*p* > 0.05) from those in adzuki bean, except that the AID of cysteine and arginine in broad beans was greater than that in adzuki bean (*p* < 0.001). The mean AID of the indispensable AA in kidney bean were the lowest (*p* < 0.001) among the values obtained for all of the cooked pulses. The mean AID of methionine and tryptophan were not significantly different (*p* > 0.05) among the pulses.

### 3.3. Mean TID of CP and AA in the Six Cooked Pulses

The TID of CP in peas was greater (*p* < 0.001) than that in kidney bean, mung bean and adzuki bean (Table 5). The mean TID of indispensable AA in broad beans and peas were greater than the values for kidney bean and mung bean (*p* < 0.001). No difference (*p* > 0.05) was observed in the mean TID of most AAs between broad beans, adzuki bean and peas, except that the TID of cysteine in broad beans and peas was greater than that for adzuki bean (*p* < 0.001), and the TID of histidine in adzuki bean was greater than that for peas (*p* < 0.001). The mean TID of methionine was not different (*p* > 0.045) among the pulses. 

### 3.4. DIAAS for the Six Cooked Pulses

For children (6 months to 3 years), the most limiting AA in cooked kidney bean, mung bean, broad beans, peas and chickpeas was lysine, and in cooked adzuki bean was leucine, though lysine was closely second-limiting. The DIAAS values (%) were: 77 for kidney bean, 68 for mung bean, 67 for chickpeas, 59 for adzuki bean, 57 for peas and 53 for broad beans. For older children, adolescents and adults, the most limiting AA in cooked kidney bean, peas and chickpeas was lysine, and in cooked mung bean, adzuki bean and broad beans was leucine. The DIAAS values (%) were: 88 for kidney bean, 86 for mung bean, 76 for chickpeas, 68 for peas, 64 for adzuki bean and 60 for broad beans (Table 6). 

The most limiting AA (the AA having the lowest ratio of dietary content to the required amount, reference protein) in the six cooked pulses was different according to the reference indispensable amino acid profile chosen for calculation of DIAAS. The DIAAS values were also different.

## 4. Discussion

According to the Food and Agriculture Organization of the United Nations (FAO) [27], a pulse is a legume that is exclusively harvested for the dry grain and therefore excludes legumes such as peanuts and soybeans, which are harvested primarily for their oil. Pulses are also sometimes referred to as grain legumes or pulse grains. The published literature often refers to pulses as including kidney bean, mung bean, adzuki bean, broad beans, peas, chickpeas, cowpeas, hyacinth bean and lentils. The pulses are the major sources of dietary fiber, minerals and vitamins for many individuals, and have the potential to meet 10–20% of the recommended daily amount of certain nutrients for adults [4,5,28,29]. Additionally, they contain significant amounts of protein (22–24% by weight) and reflect important plant-based sources of this macronutrient [4,5,28,29]. In 2017, the world area of harvested beans, was 36.5 million ha, and the production of dry beans was 31.4 million tons. The production share of beans (dry by region) was: Asia 49.3%, Americas 25.2% and Africa 21.8%. Among them, India’s output is the first (6.4 million tons) and China’s is the fourth (1.3 million tons) [1]. China has both temperate and subtropical zones and pulses comprise an important part of the agricultural production of China [2]. 

Broad beans (*Vicia faba* L.), adzuki bean (*Vigna angularis Ohwi & Ohashi*), mung bean (*Vigna radiate* L.) and kidney bean (*Phaseolus vulgaris* L.) are all mainly distributed in Asia [1,2]. China ranks first, first, second and third in the production of broad beans, adzuki bean, mung bean and kidney bean in the world [1,2]. The annual production of them in China is about 1.8 million, 1 million, 0.4 million and 0.8 million tons, respectively [1,2]. Peas (*Pisum sativum* L.) are mainly cultivated in Canada, the Russian Federation and China [1]. Chickpeas (*Cicer arietinum* L.) are mainly cultivated in India [1]. All these pulses are widely consumed in China. Their production also plays an important part in the west semi-arid and arid areas of China. Recent investigations show that the consumption of pulses is beneficial, as they reduce the risk of acquiring chronic diseases [30,31,32,33,34]. Pulses often provide a high proportion of the dietary protein for humans especially in developing countries [6]. 

An accurate assessment of the dietary protein quality and amino acid digestibility of food products is necessary [7]. DIAAS has been recommended by the Food and Agriculture Organization of the United Nations as the preferred method for determining protein nutritional quality [9,10]. To calculate DIAAS, it is necessary to determine the true ileal digestibility of each AA, which is preferably determined in humans, but if this is not possible, TID can be determined in the growing pig [13,35]. The growing pig model provides a method that allows TID to be determined in foods routinely.

AA digestibility determination at the terminal ileum is more accurate than the traditional total tract (fecal) method [35]. Although ileal digestibility may not be a perfect measure to determine net amino acid absorption, it is considerably better than the amino acid digestibility determined over the total digestive tract [36]. Dietary protein evaluation can be improved by determining the TID values of AA where TID values have been corrected for the influence of the basal gut endogenous losses [25].

In different physiological and age stages, the amounts of amino acids required daily are different. Based on the recommended amino acid scoring patterns for infants, children, older children, adolescents and adults given in the Report of the most recent FAO Expert Consultation [17], the amino acids required in the highest amounts are leucine (96 mg/g protein requirement) and then the aromatic AA (phenylalanine + tyrosine) (94 mg/g protein requirement) for infants, leucine (66 and 61 mg/g protein requirement) and lysine (57 and 48 mg/g protein requirement) for children and older children, adolescents and adults, respectively.

The most limiting AA in all six cooked pulses differed according to which amino acid reference profile was used. As expected, the DIAAS values were also different. The presently determined DIAAS values for cooked pulses ranged from 36% for broad beans to 53% for mung beans (birth to 0.5-year-old infants reference pattern), from 53% for broad beans to 77% for kidney beans (0.5 to 3-year-old child reference pattern) and from 60 for broad beans to 88% for kidney beans (older children, adolescents and adults reference pattern) (Table 6). The DIAAS values for the infant were particularly low, though it is recognized that pulses are unlikely to be sole foods for this grouping.

For children aged 0.5 to 3-year-old, the DIAAS values of cooked broad beans, peas and chickpeas were similar to values reported previously [4,5]. Kidney beans, however, had a higher DIAAS value than that previously published [4]. In addition, the first limiting amino acid, determining the DIAAS value, was different from previous reports [4,5,29]. The first limiting amino acid determining the DIAAS value of adzuki beans was demonstrated to be leucine, while for the other pulses in the present study the first-limiting AA was lysine. In previously reported studies the first-limiting AA was methionine + cysteine. Variation in DIAAS values may be a result of differences in the animal model used, analytical differences especially for the sulfur AA’s, the means of cooking the beans, or factors such as processing, bean variety, growing conditions of the pulses and the presence of anti-nutritional factors in the pulses [4,5,29]. 

Based on the cut-off value for DIAAS from the Report of the FAO Expert Consultation [9], the cooked kidney beans, mung beans and chickpeas are considered “good” protein sources for human consumption because their DIAAS is 88%, 86% and 76% (based on older children, adolescents and adults reference pattern). However, based on the 0.5 to 3-year-old child reference pattern, only kidney beans would be considered a “good” protein source.

From the current research and previous reports [4,5,29,37,38], it is concluded that the range of DIAAS values for pulses according to recommended amino acid scoring patterns for children (0.5 to 3-year-old) [9] is generally 55–83%, which is lower than that for animal proteins [9,24,25,37], but higher than that for cereal proteins [8,9,24,25,26,37,38]. The DIAAS value of animal proteins is generally higher than 90% [9,24,25,37]. However, most of the DIAAS values for cereal proteins are less than 55% [8,9,24,25,26,37,38]. Only in the work of Cervantes–Pahm et al. [26] were higher DIAAS values of 77% and 64% observed for dehulled oats and polished white rice, respectively. In the Han et al. [8] report, the DIAAS value of buckwheat was 68%. A novel aspect of the present work is that the materials were evaluated in their cooked form, as eaten by Chinese people. Cooking may alter nutritional quality.

In China, the main consumers of these cooked pulses are not infants or children aged 0.5 to 3-year-old, but older children, adolescents and adults. Currently, Dietary Guidelines for Chinese Residents recommend 250–400 g of cereal and potato-based foods per day, including 50–150 g of whole grains and edible beans. High-quality animal protein is often accompanied by high amounts of animal fat, while pulses have high-quality protein and a low amount of oil. For this reason, pulses are a good dietary choice. At the same time, when pulses are the main source of dietary energy and protein, protein complementation may be needed. Judicious combinations of pulses, cereals and relatively small amounts of other high-quality proteins can be used to maximize the supply of utilizable protein.

## 5. Conclusions

DIAAS values obtained for cooked Chinese pulses provide comprehensive nutritional information and a scientific basis for the evaluation of the nutritional values of proteins contained in different diets. The data from this study will enrich the global database of DIAAS [39], and particularly for foods relevant to Asia. The DIAAS values can be used to formulate balanced diets for humans, and inform a rational complementation of cereal proteins with pulses.

## Figures and Tables

**Table 1:** Ingredient composition of the experimental diets (g/100g as-fed basis).

Composition	Kidney Bean	Mung Bean	Adzuki Bean	Broad Beans	Peas	Chickpeas	N-Free
Cooked Kidney bean	36.8						
Cooked mung bean		38.0					
Cooked adzuki bean			41.9				
Cooked broad Beans				31.2			
Cooked peas					37.5		
Cooked chickpeas						35.7	
Corn starch	42.2	41.0	37.2	47.9	41.5	43.3	78.0
Sucrose	10.0	10.0	10.0	10.0	10.0	10.0	10.0
Corn oil	5.0	5.0	5.0	5.0	5.0	5.0	5.0
Purified cellulose	3.0	3.0	3.0	3.0	3.0	3.0	3.0
Calcium carbonate (limestone)	0.4	0.3	0.6	0.5	0.3	0.3	0.2
Calcium monophosphate	1.5	1.6	1.2	1.3	1.6	1.6	2.3
Sodium chloride	0.3	0.3	0.3	0.3	0.3	0.3	0.3
Potassium carbonate							0.3
Magnesium oxide							0.1
Titanium dioxide	0.3	0.3	0.3	0.3	0.3	0.3	0.3
Vitamin–micromineral Premix ^†^	0.5	0.5	0.5	0.5	0.5	0.5	0.5
Total	100.0	100.0	100.0	100.0	100.0	100.0	100.0

^†^ The vitamin–micromineral premix provided the following quantities of vitamins and micro minerals per kg of complete diet: Vitamin A, 5514IU; vitamin D3, 2200IU; vitamin E, 64IU; vitamin K3, 2.2 mg; thiamin, 1.5 mg; riboflavin, 4 mg; pyridoxine, 3 mg; vitamin B12, 27.6 ug; D-pantothenic acid, 14 mg; niacin, 30 mg; folic acid, 0.7 mg; biotin, 44 ug; choline chloride, 400 mg; Cu, 100 mg as cupric sulfate pentahydrate; Fe, 100 mg as ferrous sulphate monohydrate; I, 0.3 mg as potassium iodide; Mn, 40 mg as manganese oxide; Se, 0.3 mg as sodium selenite; and Zn, 75 mg as zinc oxide.

**Table 2:** Protein and amino acid compositions ^1^ of the experimental diets.

Items	Diets						
	Kidney Bean	Mung Bean	Adzuki Bean	Broad Beans	Peas	Chickpeas	N-Free
DM (%)	92.0	92.3	92.4	90.8	90.8	90.4	89.4
Crude protein (%)	9.79	9.99	9.92	9.57	9.38	9.18	0.44
Indispensable amino acids (%)							
His	0.35	0.33	0.36	0.30	0.29	0.29	0.02
Ile	0.38	0.36	0.37	0.35	0.32	0.34	0.02
Leu	1.08	1.03	1.06	0.99	0.90	0.92	0.04
Lys	0.60	0.59	0.62	0.56	0.57	0.52	0.01
Met	0.11	0.10	0.13	0.07	0.08	0.11	0.01
Cys	0.22	0.15	0.24	0.21	0.24	0.25	0.02
Phe	0.37	0.40	0.38	0.29	0.29	0.36	0.02
Tyr	0.13	0.18	0.14	0.18	0.15	0.14	0.01
Thr	0.37	0.29	0.31	0.32	0.30	0.29	0.01
Trp	0.09	0.08	0.08	0.06	0.06	0.09	-
Val	0.46	0.44	0.44	0.40	0.37	0.36	0.03
Total	4.16	3.95	4.13	3.73	3.57	3.67	0.19
Dispensable amino acids (%)							
Ala	0.40	0.38	0.38	0.39	0.36	0.36	0.02
Asp	1.02	1.02	1.02	0.97	0.93	0.97	0.03
Arg	0.46	0.57	0.55	0.87	0.78	0.76	0.01
Glu	1.44	1.53	1.53	1.54	1.48	1.42	0.05
Gly	0.35	0.33	0.34	0.37	0.36	0.33	0.01
Pro	0.24	0.27	0.30	0.29	0.27	0.28	0.03
Ser	0.52	0.42	0.44	0.42	0.38	0.40	0.01
Total	4.43	4.52	4.56	4.85	4.56	4.52	0.16
Total amino acid (%)	8.59	8.47	8.69	8.58	8.13	8.19	0.35

^1^ Determined, % as fed.

**Table 3:** Nutrient composition ^1^ of the pulses (as-fed basis).

	Ingredient					
	Kidney Bean	Mung Bean	Adzuki Bean	Broad Beans	Peas	Chickpeas
DM (%)	94.1	96.8	97.2	93.1	92.9	90.4
Crude protein (%)	26.2	26.1	25.5	29.7	24.3	25.3
Ca (%)	0.14	0.10	0.09	0.15	0.08	0.13
P (%)	0.51	0.42	0.56	0.68	0.38	0.42
Indispensable amino acids (%)						
His	0.90	0.82	0.73	0.81	0.72	0.65
Ile	1.08	1.01	0.77	1.05	0.90	0.84
Leu	1.97	1.88	2.18	2.90	1.70	2.27
Lys	1.68	1.65	1.32	1.68	1.58	1.31
Met	0.27	0.33	0.31	0.21	0.21	0.31
Cys	0.50	0.62	0.38	0.67	0.63	0.68
Phe	1.38	1.44	0.78	0.83	0.78	0.89
Tyr	0.62	0.52	0.40	0.58	0.44	0.38
Thr	1.01	0.80	0.66	0.93	0.81	0.73
Trp	0.25	0.24	0.22	0.21	0.20	0.22
Val	1.25	1.18	0.92	1.14	0.98	0.86
Total	10.9	10.5	8.67	11.0	8.95	9.14
Dispensable amino acids (%)						
Ala	1.03	1.05	0.82	1.15	1.01	0.92
Asp	2.95	2.76	2.18	2.94	2.61	2.37
Arg	1.46	1.63	1.28	2.75	2.27	1.99
Glu	3.80	4.16	3.19	4.42	3.88	3.50
Gly	0.96	0.92	0.76	1.13	1.02	0.86
Pro	0.67	0.76	0.62	0.84	0.73	0.71
Ser	1.31	1.16	0.90	1.14	0.96	1.06
Total	12.2	12.4	9.75	14.4	12.5	11.4
Total amino acids (%)	23.1	22.9	18.4	25.4	21.4	20.6

^1^ Determined values.

**Table 4:** Mean apparent ileal digestibility (AID) of crude protein (%) and amino acids (%) in the cooked pulses.

	Kidney Bean	Mung Bean	Adzuki Bean	Broad Beans	Peas	Chickpeas	SEM	*p*
Crude protein	62.7 ^c^	68.9 ^bc^	66.4 ^bc^	72.3 ^a^	77.3 ^a^	72.7 ^ba^	2.03	<0.001
Indispensable amino acids								
His	41.3 ^d^	52.0 ^dc^	74.1 ^a^	67.3 ^ba^	57.1 ^bc^	54.8 ^bcd^	3.85	<0.001
Ile	74.5 ^c^	77.4 ^c^	83.1 ^ba^	86.3 ^a^	82.2 ^ba^	79.0 ^bc^	1.31	<0.001
Leu	85.8 ^c^	87.6 ^bc^	91.0 ^a^	92.4 ^a^	89.6 ^ba^	87.0 ^bc^	0.83	<0.001
Lys	79.5 ^c^	82.0 ^c^	86.0 ^ab^	89.0 ^a^	86.8 ^ab^	83.0 ^bc^	1.11	<0.001
Met	76.4	74.7	76.8	71.7	78.3	79.0	1.76	0.078
Cys	30.2 ^b^	32.7 ^b^	40.5 ^b^	53.7 ^a^	62.7 ^a^	65.3 ^a^	3.60	<0.001
Phe	73.2 ^b^	79.6 ^a^	84.9 ^a^	85.6 ^a^	81.5 ^a^	80.7 ^a^	1.48	<0.001
Tyr	49.1 ^c^	69.8 ^b^	75.8 ^ba^	81.6 ^a^	74.7 ^ba^	66.4 ^b^	2.45	<0.001
Thr	66.8 ^b^	66.8 ^b^	77.4 ^a^	80.3 ^a^	73.6 ^ba^	69.6 ^b^	1.91	<0.001
Trp	65.2	68.1	62.9	65.3	69.0	74.2	2.68	0.082
Val	73.9 ^c^	76.1 ^bc^	82.5 ^a^	83.7 ^a^	79.2 ^ba^	75.3 ^bc^	1.51	<0.001
Mean	65.0 ^c^	69.7 ^b^	75.9 ^a^	77.9 ^a^	75.9 ^a^	74.0 ^ba^	1.58	<0.001
Dispensable amino acids								
Ala	59.0 ^c^	61.6 ^c^	75.2 ^ba^	79.5 ^a^	72.3 ^ba^	67.9 ^bc^	2.65	<0.001
Asp	85.6 ^b^	87.5 ^b^	91.4 ^a^	93.0 ^a^	90.7 ^a^	90.5 ^a^	0.70	<0.001
Arg	79.2 ^d^	84.1 ^c^	89.1 ^b^	94.6 ^a^	93.0 ^a^	92.6 ^a^	1.34	<0.001
Glu	81.6 ^d^	84.3 ^dc^	88.7 ^ba^	91.5 ^a^	88.1 ^ba^	86.6 ^bc^	1.05	<0.001
Gly	26.7 ^b^	33.0 ^b^	63.4 ^a^	68.9 ^a^	57.6 ^a^	55.3 ^a^	6.27	<0.001
Ser	76.1 ^c^	75.9 ^c^	81.9 ^ba^	85.3 ^a^	80.1 ^bc^	77.7 ^bc^	1.38	<0.001
Mean	58.3 ^b^	63.5 ^b^	73.8 ^a^	79.1 ^a^	75.1 ^a^	72.8 ^a^	2.06	<0.001

^a,b,c,d^ Mean values (*n* = 6) in a row with different superscript letters are significantly different (*p* < 0.001).

**Table 5:** Mean true ileal digestibility (TID) of crude protein (%) and amino acids (%) in the cooked pulses.

	Kidney Bean	Mung Bean	Adzuki Bean	Broad Beans	Peas	Chickpeas	SEM	*p*
Crude protein	76.0 ^c^	81.9 ^bc^	79.6 ^bc^	85.7 ^ba^	90.9 ^a^	86.6 ^ba^	2.03	<0.001
Indispensable amino acids								
His	56.5 ^d^	68.1 ^c^	88.9 ^a^	84.9 ^ba^	75.2 ^bc^	72.6 ^bc^	3.85	<0.001
Ile	79.9 ^d^	83.0 ^dc^	88.6 ^ba^	92.0 ^a^	88.4 ^ba^	84.8 ^bc^	1.31	<0.001
Leu	88.7 ^c^	90.6 ^bc^	93.9 ^a^	95.5 ^a^	93.0 ^ba^	90.3 ^bc^	0.83	<0.001
Lys	83.6 ^d^	86.1 ^cd^	89.9 ^abc^	92.9 ^a^	91.0 ^ab^	87.6 ^bc^	1.11	<0.001
Met	84.2	83.1	83.4	83.3	88.5	86.7	1.76	0.186
Cys	43.7 ^b^	53.0 ^b^	52.9 ^b^	67.8 ^a^	75.2 ^a^	76.9 ^a^	3.60	<0.001
Phe	77.8 ^c^	83.9 ^b^	89.4 ^ba^	91.4 ^a^	87.3 ^ba^	85.3 ^b^	1.48	<0.001
Tyr	59.1 ^c^	76.8 ^b^	85.1 ^ba^	88.5 ^a^	82.8 ^ba^	75.7 ^b^	2.45	<0.001
Thr	74.6 ^c^	76.9 ^bc^	86.6 ^a^	89.2 ^a^	83.1 ^ba^	79.4 ^bc^	1.91	<0.001
Trp	77.9	82.2	77.0	83.5	87.2	86.9	2.68	0.045
Val	79.9 ^c^	82.8 ^bc^	89.2 ^a^	91.1 ^a^	87.1 ^ba^	83.4 ^bc^	1.51	<0.001
Mean	73.3 ^c^	78.8 ^b^	84.1 ^ab^	87.3 ^a^	85.3 ^a^	82.7 ^ba^	1.58	<0.001
Dispensable amino acids								
Ala	70.3 ^d^	73.4 ^dc^	87.0 ^ba^	90.8 ^a^	84.4 ^ba^	80.1 ^bc^	2.65	<0.001
Asp	88.0 ^b^	89.9 ^b^	93.8 ^a^	95.4 ^a^	93.0 ^a^	92.8 ^a^	0.70	<0.001
Arg	84.0 ^c^	88.1 ^b^	93.1 ^a^	97.2 ^a^	96.1 ^a^	95.5 ^a^	1.14	<0.001
Glu	85.8 ^d^	88.2 ^dc^	92.6 ^ba^	95.4 ^a^	92.1 ^ba^	90.8 ^bc^	1.53	<0.001
Gly	47.1 ^b^	54.6 ^b^	84.3 ^a^	87.8 ^a^	76.9 ^a^	76.2 ^a^	6.27	<0.001
Ser	81.7 ^c^	82.7 ^c^	88.4 ^ba^	92.1 ^a^	87.5 ^ba^	84.8 ^bc^	1.38	<0.001
Mean	76.1 ^b^	79.5 ^b^	89.9 ^a^	93.1 ^a^	88.4 ^a^	88.7 ^a^	2.01	<0.001

^a,b,c,d^ Mean values (*n* = 6) in a row with different superscript letters are significantly different (*p* < 0.001). TID values were calculated by correcting the values of apparent ileal digestibility for the basal endogenous losses. Values used for the basal endogenous losses were as follows (g/kg DMI): Asp: 0.45; Ser: 0.27; Glu: 0.57; Gly: 0.67; His: 0.50; Arg: 0.21; Thr: 0.27; Ala: 0.42; Pro: 3.75; Cys: 0.28; Tyr: 0.12; Val: 0.28; Met: 0.08; Reactive-Lys: 0.22; Lys: 0.23; Ile: 0.19; Leu: 0.29; Phe: 0.16; and Trp: 0.11.

**Table 6:** Digestible indispensable amino acid scores (DIAAS) for the cooked pulses.

	Kidney Bean	Mung Bean	Adzuki Bean	Broad Beans	Peas	Chickpeas
DIAA ratio (infants (birth to 6 months))						
His	2.14	2.32	1.70	1.96	2.21	2.22
Ile	0.58	0.55	0.44	0.41	0.45	0.55
Leu	0.58	0.55	0.40	0.38	0.44	0.55
Lys	0.61	0.61	0.50	0.43	0.48	0.53
SAA	1.09	1.00	1.32	1.24	1.19	1.29
AAA	0.50	0.53	0.37	0.36	0.41	0.49
Thr	0.85	0.74	0.61	0.64	0.73	0.78
Trp	0.60	0.57	0.60	0.53	0.47	0.59
Val	0.73	0.72	0.55	0.54	0.60	0.67
DIAAS (%)	50 (AAA)	53 (AAA)	37 (AAA)	36 (AAA)	41 (AAA)	49 (AAA)
DIAA ratio (child (6 months to 3 years))						
His	2.25	2.43	1.79	2.05	2.32	2.33
Ile	0.99	0.95	0.75	0.70	0.77	0.94
Leu	0.85	0.79	0.59	0.55	0.64	0.81
Lys	0.74	0.74	0.60	0.53	0.58	0.64
SAA	1.33	1.22	1.61	1.51	1.46	1.57
AAA	0.91	0.95	0.66	0.66	0.74	0.89
Thr	1.21	1.05	0.86	0.91	1.03	1.10
Trp	1.20	1.14	1.21	1.06	0.95	1.18
Val	0.94	0.92	0.70	0.70	0.77	0.85
DIAAS (%)	77 (Lys)	68 (Lys)	59 (Leu)	53 (Lys)	57 (Lys)	67 (Lys)
DIAA ratio (older child, adolescent, adult)						
His	2.81	3.04	2.24	2.57	2.90	2.91
Ile	1.05	1.02	0.80	0.75	0.82	1.00
Leu	0.92	0.86	0.64	0.60	0.70	0.87
Lys	0.88	0.88	0.71	0.62	0.68	0.76
SAA	1.56	1.44	1.89	1.78	1.71	1.85
AAA	1.15	1.21	0.84	0.84	0.94	1.13
Thr	1.50	1.31	1.07	1.13	1.28	1.37
Trp	1.55	1.47	1.55	1.37	1.22	1.52
Val	1.01	0.99	0.75	0.75	0.82	0.92
DIAAS (%)	88 (Lys)	86 (Leu)	64 (Leu)	60 (Leu)	68 (Lys)	76 (Lys)

First limiting amino acid is given in brackets. SAA, Sulphur amino acids. AAA, aromatic amino acids.

## Abbreviations

DIAAS	digestible indispensable amino acid score
AA	Amino acid
CP	crude protein
TID	true ileal digestibility
TIDAA	true ileal digestibility of AA
AID	apparent ileal digestibility
AIDAA	apparent ileal digestibility of AA
DM	Dry matter
DMI	Dry matter intake
FAO	Food and Agriculture Organization of the United Nations
SAA	Sulphur amino acids
AAA	aromatic amino acids
His	Histidine
Ile	Isoleucine
Leu	Leucine
Lys	Lysine
Met	Methionine
Cys	Cystine
Phe	Phenylalanine
Tyr	Tyrosine
Thr	Threonine
Trp	Tryptophan
Val	Valine
Ala	Alanine
Asp	Aspartic acid
Arg	Arginine
Glu	Glutamic acid
Gly	Glycine
Ser	Serine
